# Effects of *Spirulina platensis* supplementation on lipid profile in HIV–infected antiretroviral naïve patients in Yaounde - Cameroon: a randomized trial study

**DOI:** 10.1186/1476-511X-13-191

**Published:** 2014-12-13

**Authors:** Marthe-Elise Ngo-Matip, Constant Anatole Pieme, Marcel Azabji-Kenfack, Prosper Cabral Nya Biapa, Nkenfack Germaine, Englert Heike, Bruno Moukette Moukette, Korosky Emmanuel, Stefanini Philippe, Carl Moses Mbofung, Jeanne Yonkeu Ngogang

**Affiliations:** National Institute of Agro-Industrial Sciences, University of Ngaoundere, POBOX 455, Ngaoundere, Cameroun; Department of Physiological Sciences and Biochemistry, Faculty of Medicine and Biomedical Sciences, University of Yaounde 1, POBOX 1634, Yaounde, Cameroon; Charity Health Centre, Berlin, Germany; Department of Biochemistry, University of Dschang, Dschang, Cameroon; Spirulina Producer’s Federation of France, Hyères, France; Ecole de formation Agricole de Hyères, Hyères, France

**Keywords:** Spirulina platensis, Atherosclerosis, HIV+ antiretroviral naïve, Lipid profile

## Abstract

**Background:**

Cardiovascular diseases (CVD) and metabolic alterations are among the majors public health concern that have been reported in people living with HIV infections. Factors contributing to cardio metabolic syndrome in HIV include body fat distribution, dyslipidemia, insulin resistance, cardiovascular dysfunction and inflammation. The aim of the study was to determine the effect of *Spirulina platensis* (Cyanobacteriaceae) supplementation versus local diet on lipid profile in HIV-infected antiretroviral-naive patients.

**Methods:**

A prospective single-blind, randomized, multicentre study was conducted from February 2010 to December 2012. A total of 320 HIV antiretroviral-naïve patients were screened and 169 were recruited in this study. Patients were randomized and received either Spirulina supplementation combined with local diet (n = 82) or local diet only (n = 87). Age, weight, body mass index (BMI), lipid profile, CD4 count, and local food intake variables were assessed on three separate occasions (three, six and twelve months).

**Results:**

An average age of the patients was 35.6 ± 9 years. The majority of participants were female 67.1%. Regarding the lipid profile, there is a significant increase in HDL-cholesterol and a significant decrease in total cholesterol, LDL-cholesterol and triglycerides in the group of patients who consumed *Spirulina platensis*. A change in the atherogenic index defined by the ratio CT/HDL-C substitutable by LDL-C/HDL-C and the TC/HDL decreased significantly from 10.83 at baseline to 2.22 after 12 months (p = 0.21 and p <0.0001) in the patients taking Spirulina.

**Conclusions:**

Nutritional supplementation with Spirulina combined with a quantitative and qualitative balanced diet for at least six months can retard an exposition to lipid abnormalities in HIV–infected antiretroviral-naive patients. Further studies are recommended on a large group of people not infected with HIV and exposed to cardiovascular risk factors.

## Background

Patients with human immunodeficiency virus (HIV) infection are at increased risk of developing coronary heart disease (CHD) although traditional risk factors contribute to elevate the risk [1]. There is growing evidence that HIV-infected individual develop more risk of coronary heart disease (CHD) than healthy patient. Studies showed that the incidence of the CHD risk of HIV-infected patient varied from different countries [2]. Recent reports have shown that cardiovascular deaths accounted for 6.5% of total deaths in a large observational cohort of HIV-infected patients from Europe and North America, for 8% of deaths among HIV-infected people in France, and for 15% of deaths in a North American HIV outpatient study [3–6]. In Sub-Saharan Africa, one of the several Public Health problems is HIV/AIDS [7]. Since the early days of the HIV epidemic, the effects of HIV disease on serum lipids have been described [8]. Cardiovascular diseases have been the leading cause of morbidity and mortality in the general population [3] and have now also been identified as a major cause of death in people with HIV/AID [9].

Cardiovascular diseases (CVD) have emerged as a major public health problem and impose an escalating burden on the health care system in Cameroon. A study in this country between 1992 and 1997 ranked coronary artery disease eighth among the CVDs registered with a prevalence of 1.53 percent [10, 11]. A study conducted between November 2009 and November 2011, in a population of 8,389 adults and 706 children consulted in the St. Elizabeth Catholic General Hospital of Shisong, Cameroon showed the increase of incidence with 41.5% diagnosed for Hypertension; 29.6% for congestive heart failure, 12.2% for arrhythmia [12]. Data from the same study demonstrated and confirmed previous works which showed that cardiac involvements in human immunodeficiency virus (HIV) infection, with cor pulmonale and pericarditis contribute to over 20% of cases of heart failure [12, 13]. In Cameroon, about 5.5% of the population are infected of HIV mostly women and individuals aged between 15 and 49 years are most commonly identified [14]. Studies reported an increase of triglycerides (TG), total cholesterol (TC), low density lipoprotein cholesterol (LDL-C), and decrease of high density lipoprotein cholesterol (HDL-C) has raised concern about increased risk of atherogenesis and atherosclerotic vascular disease [15–18]. These abnormalities are common in adults infected with the Human Immunodeficiency Virus (HIV) [19]. The HIV infection itself has been shown to cause changes in glucose and lipid metabololism [20, 21]. Few studies have been carried out in Cameroon on disturbances in lipid metabolism and nutritional deficiency in HIV-infected patients [22]. In spite of the relatively high prevalence of HIV in this country, little is known about cardiovascular disease (CVD) among HIV-infected antiretroviral naive persons [8]. There are numerous evidences that nutritional deficiency (ND) is highly prevalent in HIV-infected patients, especially among patients from Sub Saharan Africa [23]. The risk factors of Nutritional Deficiency in HIV infected patients are: a precarious social situation, poor adherence to diet modification and comorbidities. Very few researches on the effects of dietary supplements combined with an appropriate balance diet and medical follow up of HIV infected patients have been published. The dietary supplements strategy could be in the long period slow down the progression of metabolic complications among HIV-infected antiretroviral naïve patients. Due to the difficulties to access to a balanced diet, a supplementation of a safety cyanobacterium such as *S. platensis* in the diet or in the treatment of HIV patients naives or under antiretroviral drugs is necessary [24]. This blue green alga is used as a food supplement all over the world [25, 26]. Studies have demonstrated its beneficial effects on human health on glucose, lipid metabolism and blood pressure through its full content in antioxidant combined with vitamin A, B12, E, proteins and mineral salt and also in building immunity of patients with HIV infection and multiple cardiovascular risk factors [24, 27–29]. It also was reported that the protein C-phycocyanin present in *S. platentis* play a crucial role in the decrease of hypercholesterolemic action [30, 31].

The determination of lipid parameters is not required during follow-up of HIV-infected patients. Although disturbances in lipid metabolism have been found in HIV-infected patients, no study has yet been carried out to determine whether these disturbances are caused by the treatment or by other factors. However, a previous study in Cameroon has shown its interest in severely infected people living with HIV [24].

The present study was designed to assess the potential effect of *S. platensis* supplementation on the progression of atherogenetic dyslipidaemia among HIV-Infected antiretroviral naïve patients.

## Methods

### Nutritional composition of *S. platensis*

Dry S. *platensis* powder was obtained from a local farm of spirulina in Central Africa. It was kindly donated by a Cameroonian Association of Knowledge-Attitudes-Practices “KAP” in Yaounde-Cameroon. The Table 1 shows it’s the composition (g per 100 grams of powder). The total protein was determined by using the Kjeldahl method [32]. The total lipids, dietary fiber and ash were determined using the standard methods of AFNOR [33]. The total sugar was determined by using standard methods of Cerning and Guilbot [34] while the minerals salt were described by using the AOAC [32].

**Table 1:** **Composition of freeze-dried powder of** ***Spirulina platensis*** **intake in our study**

Composition	g/100 of dry mass (%)	Energetic values (kilo calories)
**Macronutrients:**		
**Total Proteins (g)**	61.81	258.36
**Lipids (g)**	16.06	144.54
**Carbohydrates (g)**	12.36	51.66
**AHK (g)**	9.77	
**Dry mass**	88.75	
**Dietary fiber (g)**	02.26	
**Micronutrients**		
**Calcium (mg)**	0.28	
**Magnesium (mg)**	0.93	
**Potassium (mg)**	14.40	
**Na + (mg)**	1.96	
**Total Nitrogen**	90.75	
**Iron (mg)**	21.98	
**pH**	7.81	

The analysis of the freeze-dried spirulina powder sample obtained from the Institute of Medical and Medicinal Plants (IMMP) (Yaounde-Cameroon).

### Criteria of selection of population of study

We carried out a longitudinal study in a randomized cohort from January 2011 to February 2012. Patients aged between 18 – 65 years, HIV–infected antiretroviral naïve to treatment and with CD4 count ≥400 cells/μL were eligible. Patients who shown their CD4 drop to ≤400 cells/μL during the follow-up were excluded. Participants were also required not to be on lipid modifying therapies at their enrolment. Informed consent was obtained from every subject prior to the participation. The study was approved by the Cameroon National Ethic Committee under the following reference number: 123/CNE/SE/2011, Cameroon. The subjects were recruited by the medical file of the Day Care Clinic of Central Hospital of Yaounde (CHY) and of the Biyem-Assi District Hospital. Investigation and intervention were carried out at the Etoug-Ebe Health Center.

### Selection, randomization, treatment allocation and follow up

**Figure 1:**
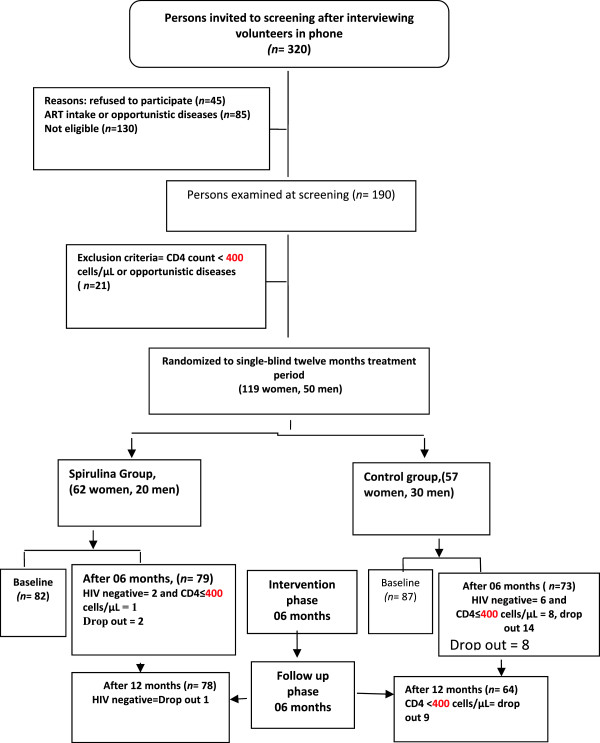
**Flow chart describing progress of participants through the** ***Spirulina platensis*** **supplementation trial.**

Selected participants (169 subjects) were included in the study among 116 women and 53 men. Of the 169 subjects, we allocated 87 subjects to the control group (first group) and 82 subjects to the intervention group (second group). HIV infected patients were selected after checking patients files for CD4 counts above 400 cells/μl who were ARV naïve to treatment (Figure 1). The two groups were well matched with respect to age, sex, and CD4 cells counts. At the entrance of the study, patients had been fasting for at least eight hours overnight.

The patients of the first group were advised to take a fresh local balance diet while patients of the second group were asked to add *S. platensis* in their local diet during the first six months. The *S. platensis* was given in package of powder doses of 10 grams per day. The next six months were for the follow-up without *S. platensis* powder. Anthropometric measurements (weight, height, BMI) were done at baseline, after six and twelve months for the two groups.

All anthropometric measurements were done with the same electronic weighing device (SECA QUALITY SEAL – GERMANY). The BMI was calculated using Quetelet’s formula; Weight/height^2^ and expressed as Kg/m^2^ to the nearest one decimal place.

Subjects were treated “as usual” “usual care” treatment in Cameroon means patients are subjected to practitioner’s choice of therapy. This consists of routine fasting blood sugar level and lipid profile control after six months. To monitor these parameters, two types of standardized questionnaire for demographic characteristics, clinical, quality of live (alcohol consumption, tabac, physical activities and dietary habits), and a three day diet intake data was filled by the subjects at the baseline, during and at the end of the trial.

### Dietary records

Food intake was recorded using a predesigned “three days” questionnaire. Subjects had to document time, quantity and quality of food or beverage consumed. They were instructed to: (1) document all consumed foods and beverages in as much detail as possible, (2) to weigh foods (or to estimate doses, if weighing was not possible in some situations), (3) to document food or beverage intake immediately after consumption and (4) not to change usual eating habits. Data analysis was performed using the nutrition software EBISpro2011, version 8.0 for Windows. In analyzing the eating records, nutrients were studied in relation to the dietary reference intakes (DRI) (EBISpro 2011) [35]. The DRI utilized corresponds to the age group and sex. The nutrients evaluated in this study include are posted in Table 2. Deficiency was defined as a concentration below the reference interval: Proteins 57.1 g, Iron 10 mg, Zinc 10 mg, Calcium 1000 mg, Potassium 3500 mg, Magnesium 350 mg, Cholesterol 300 mg.

**Table 2:** **Micronutrients intake of the HIV-ARV naïve population before and after spirulina supplementation**

Variables	Ctrol/Spi	Period	Patients received local balance diet	Patients received spirulina combined with a local balance diet	***p-value***
**Proteins (g)**	87/82	T0	60.51 ± 16.39	48.03 ± 8.85	<0.001
	79/80	T6	62.61 ± 14.12	66.03 ± 9.3	0.10
	66/79	T12	60.28 ± 13.52	72.77 ± 7.3	<0.001
**Iron (mg)**	87/82	T0	12.03 ± 1.12	12.85 ± 2.64	<0.01
	79/80	T6	11.86 ± 1.30	16.04 ± 1.20	<0.001
	66/79	T12	11.80 ± 1.14	14.67 ± 3.75	<0.001
**Zinc (mg)**	87/82	T0	81 ± 6.35	70 ± 4.50	<0.001
	79/80	T6	6.36 ± 2.67	5.79 ± 1.70	0.15
	66/79	T12	4.28 ± 3.16	6.92 ± 1.53	<0.001
**Calcium mg**	87/82	T0	487.09 ± 194.58	666.18 ± 680.75	<0.02
	80/79	T_6_	509.60 ± 248.11	1339.90 ± 303.37	<0.001
	79/66	T_12_	510.87 ± 220.48	943.85 ± 308.39	<0.001
**Potassium (mg)**	87/82	T0	1148,48 ± 2,00	766,57 ± 2,40	<0.01
	79/80	T6	2306 ± 733.53	4555 ± 798.93	<0.001
	66/79	T12	2593 ± 112.44	3241 ± 709.83	<0.001
**Magnesium (mg)**	87/82	T0	223.23 ± 114.60	250.10 ± 91.99	0.11
	79/80	T6	231.61 ± 88.78	428.44 ± 78.5	<0.001
	66/79	T12	251.21 ± 143.00	288.00 ± 80.70	0.07
**Cholesterol (mg)**	87/82	T0	379.37 ± 83.24	464.54 ± 160,74	<0.001
	79/80	T6	402.77 ± 66.29	216.32 ± 100.50	<0.001
	66/79	T12	413.88 ± 56.05	292.67 ± 55.54	<0.001

Mean values ± SD, Ctrl: Control, Spi: Spirulina group T0: initial period, T6: Six months, T12: Twelve months Mean values ± SD. *p* < 0.01: Significant difference between the group.

### Determination of biochemical parameters

The blood samples were obtained through patients in two tubes of 4 mL, one dry and the content Ethylene diamino Tetracetic Acid (EDTA). The dry tube was used to determine the lipid profil using coloring method with Kits (Human). The LDLC-C levels were determined using method of Friedewald *et al*. [36]. The following definitions were used for dyslipidemia: Total cholesterol ≥ 240 mg/dl, Low-density lipoprotein cholesterol ≥ 130 mg/dl, Triglycerides ≥150 mg/dl, and High-density lipoprotein cholesterol < 40 mg/dl. TC/HDL-C and LDLC/HDLC ratios were used to calculate the atherogenicity index. When the ratio TC/HDL-C > 4.85 and LDL-C/HDLC > 3.55 than the atherogenic index is statistically significant.

Fasting blood sugars were determined using « ULTRA ONE TOUCH” glucometer. The level of glycemia higher than ≥ 110 mg/dL were identified like cardiovascular risk factors.

The Flow Cytometry (FACS CALIBUR II Machine) was used for the determination of CD4 lymphocytes count in all patients. Values were expressed as cells/μL.

### Statistical analysis

Each experiment was carried out in triplicate. Results were considered significant at *p*-value less than 0.05. Data are presented as mean ± standard deviation or as percentage. Chi-square was performed to determine the significance of differences in the prevalence of dyslipidemia in both groups. Student’s *t*-test version 3.3.2 was used to compare the lipid parameters of both groups. The statistical SPSS program (version 16.0) was used for all the analysis.

## Results

### Dietary records

The results of the nutrients intake of the population the two groups can be classified in two groups which include macronutrients and mineral (Table 2). The results showed the increase of protein intake and the decrease of the cholesterol level with significant difference during the last 6 months of the study to the group of population who add *S. platensis* to their local diet. In contrary to this group, we found the increase of cholesterol level to the other group and the variation of protein (Table 2). Regarding the micronutrients intake, the Iron, Calcium and Magnesium increase significantly during the trial in the population who supplemented *S. platensis* in their diet (Table 2).

### Characteristics of population studied

The sample population of the study at the baseline was 169 (Figure 1). Among them, four subjects (2.36%) from the intervention group and 23 subjects from the control group (13.60%) were dropped out of the trial at the end of twelve intervention months. Six of the 27 subjects in the control group and two of three subjects in the intervention group were HIV–negative due to the error of screening test in the laboratory (Figure 1). Seventeen subjects in the control group and two subjects in the intervention group were dropped out due to a decrease in their CD4 cells count. The distribution of the 27 drop-out according to the cause of drop-out and treatment group is presented in (Figure 1).

Finally for the 142 subjects (84.01%) who demonstrated the atherogenic lipid abnormalities in HIV-infected antiretroviral-naïve patients were used to continue the study. Demographic characteristics of the 169 HIV–Infected antiretroviral naïve patients are presented in (Table 3). The results showed that this sample population was made of women (76.1%) and men (23.9%). The two groups were similar with respect to age, sex. The mean age was 35.6 ± 9 years. The value of the BMI decreases none significantly during the twelve months in both groups. The results of clinical characteristics of patients in the spirulina group (Spi) and control show that the presence of sexual transmitted infections (3%, 16%), malaria (6%, 13%) and shingles (3%, 7%). As for lifestyle patients practiced physical activity drank alcohol and tobacco smoke (Table 3). The fasting blood sugar was decreased in the group of patients who add 10 g of *S. platensis* compare to the other group which in which the variation was no linear. No significant difference was observed in fasting blood sugar in both groups at the first 6 six months. At the end of the study a significant difference was noted in these two groups (Table 3).

**Table 3:** **Baseline of demographic and clinical characteristics of patients during the trial**

Variables	Ctrl/Spi	Period	Patients received local balance diet	Patients received spirulina combined with a local balance diet	***p-value***
**Age (Years)**	87/82	T0	35.43 ± 10.04	36.01 ± 9.44	0,58
**Sex (%)**					
**Female**	87/82	T0	64.5% (56)	69.8% (60)	0.06
**Male**			35.6% (31)	25.6% (22)	
**BMI (kg/m** ^**2**^ **)**	87/82	T0	26.02 ± 4.97	25.29 ± 4.54	0.33
	79/80	T6	25.18 ± 6.41	25.41 ± 4.98	0.80
	66/79	T12	22.18 ± 1.07	23.26 ± 6.53	0.44
**Fasting blood sugar (mg/l)**	87/82	T0	115 ± 53,70	105.89 ± 25.91	0.159
	79/80	T6	105.00 ± 30.76	103.14 ± 15.93	0.65
	66/79	T12	113.77 ± 61,85	95.35 ± 9.63	<0,001
**Alcohol**	14/169	To	11.9%	11.9%	
	10/156	T6	11.9%	5.6%	
	7/145	T12	4.4%	4.4%	
**Smoking**	5/169	To	3%	4.5%	
	5/169	T6	3%	4.5%	
	6/169	T12	1.4%	3%	

Ctrl: Control, Spi: Spirulina group, T0: initial period, T6: Six months, T12: Twelve months, p< 0.01: Significant difference between the group.

### Changes in lipid profile and atherogenic index

To investigate the cardiovascular risk factors of these groups of population, the lipid profile which include the TG, TC, HDL-C, LDL-C and the artherogenic index were determined.

**Figure 2:**
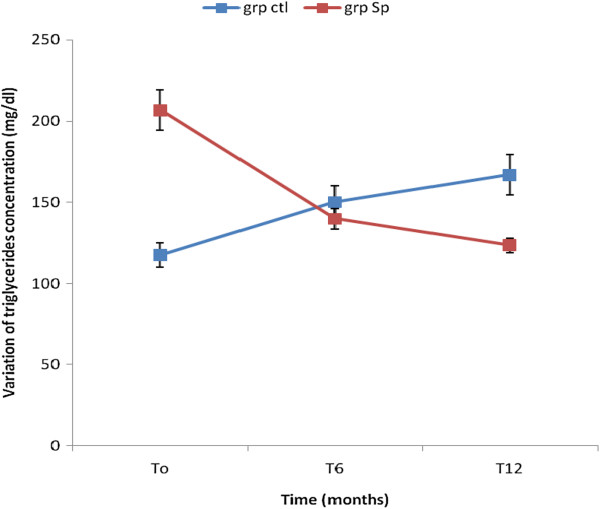
**Concentration of triglycerides levels of the two groups of patients during the trial.**

**Figure 3:**
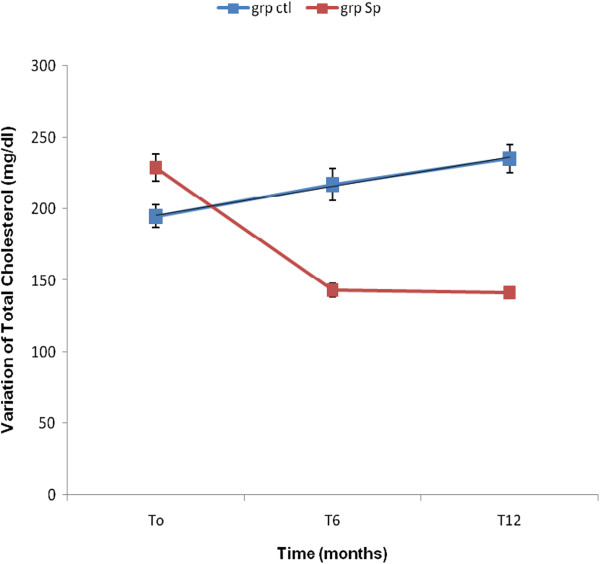
**Concentration of total cholesterol levels of the two groups during the trial.**

**Figure 4:**
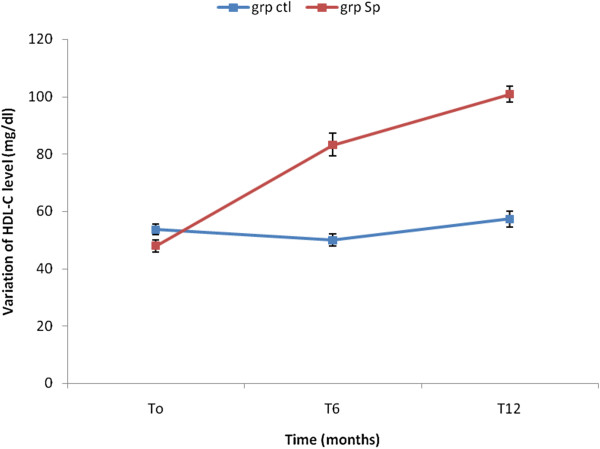
**Concentration of HDL-Cholesterol levels of the two groups during the trial.**

**Figure 5:**
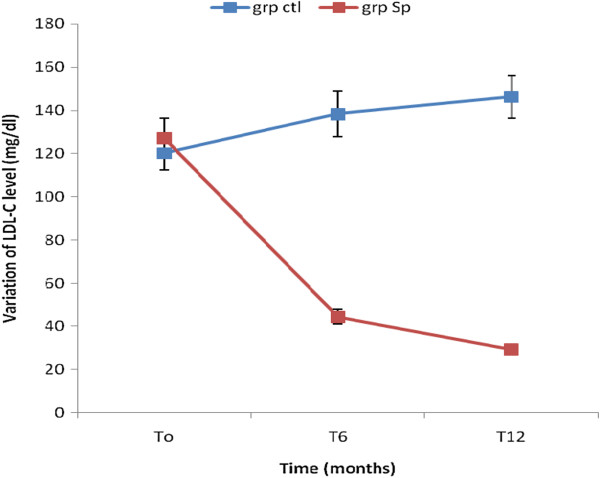
**Concentration of LDL cholesterol of the two groups during the trial.**

**Figure 6:**
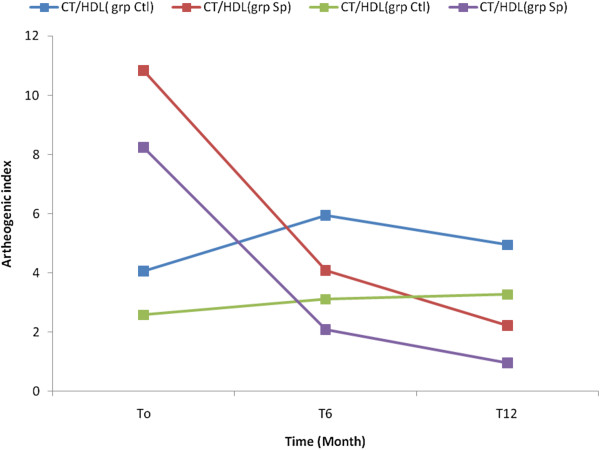
**Concentration of artherogenic index of the patients in the two groups during the trial.**

The results showed that the concentration of TG, TC and LDL-C significantly decreased in the Spi group while those of the control group increased (Table 4). This result shows that the consumption of *S. platensis* positively and significantly affects the lipids profile. The Figures 2, 3, 4, 5 and 6 show the variation of lipids profile during the experiment. This results showed that the levels of TG of patient from Spi group, initially higher than those of control group significantly dropped at the end of the intervention (206.89 versus 117.59 p < 0.0001) and (98.90 versus 162.25 p < 0.0001) (Figure 2). In the same population, the levels of HDL-C increased significantly from 48.00 to 100.98 p = 0.71, p < 001, p < 0.0001) during the same period of study (Figure 4). The results of atherogenic index defined by the ratio of TC/HDL-C or LDL-C/HDL-C showed these ratio significantly decreased from 10.83 to 2.22 after twelve month (p = 0.21, p = 0.64, p < 0.0001) for (TC/HDL-C) and from 8.24 to 0.96 (p = 0.26, p = 0.21, p < 0.0001) (LDL-C/HDL-C) for patients using *S. platensis* as supplement while for the control group these the value this ratio increased (Figure 6). These results demonstrated that the supplementation of *S. platensis* on diet have benefit effect on the lipid profile of HIV patients.

**Table 4:** **Change in lipid profile during the six months of intervention and the follow up in each group**

Variables	Ctrl/Spi	Period	Patients received local balance diet	Patients received spirulina combined with a local balance diet	***P-value***
**Total cholesterol (mg/dL)**	87/82	T0	194.8 ± 80.1	228.7 ± 96.8	0.17
	79/80	T6	216.8 ± 109.4	143.3 ± 45.4	<0.001
	66/79	T12	234.9 ± 101.8	141.4 ± 40.1	<0001
**HDL cholesterol (mg/dL)**	87/82	T0	53.6 ± 18.4	48.0 ± 21.1	0.71
	79/80	T6	49.9 ± 21.6	83.2 ± 39.7	<0. 001
	88/79	T12	57.3 ± 27.0	100.9 ± 28.7	<0.001
**LDL cholesterol (mg/dL)**	87/82	T0	120.12 ± 75.8	127.0 ± 92.2	0.60
	79/80	T6	138.3 ± 105.8	44.5 ± 33.0	<0.001
	66/79	T12	146.3 ± 97.8	29.3 ± 16.8	<0.001
**Triglycerides (mg/dL)**	87/82	T0	117.6 ± 76.8	206.9 ± 122.7	<0.0001
	79/80	T6	150.4 ± 97.5	139.8 ± 64.2	0.44
	66/79	T12	167.1 ± 124.8	123.5 ± 44.6	<0.01
**CT/HDL (mg/dL)**	87/82	T0	3.6 ± 4.3	4.7 ± 4.6	0.21
	79/80	T6	4.34 ± 5.1	1.7 ± 1.1	0.64
	66/79	T12	4.1 ± 3.0	1.4 ± 1.4	<0.0001
**LDL/HDL (mg/dL)**	87/82	T0	2.2 ± 4.1	2.6 ± 4.3	0.26
	79/80	T6	2.8 ± 4.89	0.53 ± 0.84	0.21
	66/79	T12	2.5 ± 3.6	0.3 ± 0.6	<0.0001

Mean values ± SD.

Ctrl: Control, Spi: Spirulina group; TC: HDL-C and LDL-C: HDL-C ratios were used to calculate the atherogenicity index. TC, total cholesterol; HDL-C, high-density lipoprotein cholesterol; LDL-C, low-density lipoprotein cholesterol; TG, triglycerides, P < 0.01: Significant difference between the group.

## Discussion

The present randomized control study was designed to compare the effects of consumption of *S. platensis* on some metabolic parameters among HIV–infected antiretroviral naïve patients. Studies showed that dietary supplements are often used by people living with HIV infection [37]. Previous research also demonstrated similar use, such as a study of HIV positive men that found 69% used complementary medicine products and practices, the most common of which were dietary supplements [38]. The most common dietary supplements in people living with HIV/AIDS (PLWH) are used to ‘boost immune functioning’ such as mega-dose vitamins, and anti-oxidants and body cleansing products such as teas and herbs to remove ‘toxins’ [39]. It is important to note that while some dietary supplements can adversely interact with prescription medications [40], some supplements may also have positive impacts on HIV disease processes. The supplementation of *S. platensis* in diet improves the living conditions of HIV patients by providing macronutrients and energy which play an important role on the health of these patients.

The variation of the lipid profile among HIV-infected antiretroviral naïve patients was determined in the study. Dyslipidemia is one of the significant modifiable risk factors for cardiovascular diseases. The dyslipidemia is characterized by a decrease of the levels of high-density lipoproteins cholesterol (HDL-C), an increase of the levels of low-density lipoprotein cholesterol (LDL-C) and elevated levels of triglycerides (TG). In the initial state, our study showed that the level of TC, LDL-C and TG was lower in the control compare to compare to the group of patients supplementing with *S. platensis*. Our observations are in agreement with previous studies in United States and other parts of sub-Saharan Africa that documented the lipid profile of the treatment-naïve HIV-infected individuals [41–44]. After six months, the levels of TC, LDL-C in the serum were higher in the control group compare to the group of patients supplementing with *S. platensis*. At the same time, the concentration of HDL-C increased during the same period. The increase of the cholesterol levels, in particular LDL-C, TG and TC in the control group suggest the disturbance in the lipids metabolism which can be attributed to the malnutrition [22]. This result demonstrated that *S. platensis* positively and significantly affect the dyslipidemia through the inversion of lipid profile in the group receiving *S. platensis*. The increase TG level in the serum probably caused by an increase of VLDL levels has previously been found to be linked to an increase in the synthesis of hepatic fatty acids. In our study, TC was significantly lower in patients who received *S. platensis* as supplement with a significantly higher concentration of HDL-C in the same group at the end of the trial. The elevation of the HDL-C level occurs before hypertriglyceridemia, correlates with other findings which demonstrated that supplementation of diet with S. *platensis* decreases LDL-C and increases HDL-C with a probable beneficial effect [29, 30]. Investigations on the effects of lowering blood cholesterol by *S. platensis* in rats [45, 46] and in different doses (5- 16% of diet) have been published [30]. These studies demonstrated that HIV infection induces an increase of the TG, LDL-C, TC level and the reductions of HDL-C in accordance of other studies [47, 48]. The observed alteration of cholesterol metabolism in HIV-infected patients may be explained by lipid peroxidation [22, 47]. The observed alteration of cholesterol metabolism in HIV–infected patients may be explained by the increase of lipid peroxidation through stimulating the production of reactive oxygen species [49]. Other mechanism of lipid disorders in ART-naïve HIV-infected patients is also thought to be cytokine-mediated [44]. A possible relationship between lipids profile and immune system has been noted. Constans *et al*. [50] reported that HIV infection itself induced a progressive increase in TG and LDL-C. The mechanism used by *S. platensis* to reduce the hypercholesterolemia and lipid disorders have not yet been elucidated although some authors suggest that the addition of this alga into the diet diminishes the intestinal absorption of cholesterol as well as the re-absorption of bile acids in the ileum [51, 52]. Thus, they suggest that spirulina can be considered a functional food capable of reducing the levels of cholesterol and consequently preventing atherosclerosis. The antioxidant mechanism of *S. platensis* can also be suggested due to the presence of protein C-phycocyanin, which the structure is closed to that of bilirubin which plays an important physiological role against reactive oxygen species [31, 53]. This C-phycocyanin protein inhibits oxidative changes in plasma proteins and aromatic amino acid residues [53], increases activity of the enzyme lipoprotein lipase, which is a key enzyme in the metabolism of triglycerides and lipoproteins [54]. These results showed that *S. platensis* demonstrated hypercholesterolemia effects. Although dyslipidemia in HIV-infected patients is believed to carry a similar risk as in HIV-negative populations, the consistent findings of low HDL-C in combination with hypertriglyceridemia can easily increase the burden of cardiovascular diseases in HIV-infected patients by unwanted proportions [55]. This is because hypertriglyceridemia and low HDL-C are recognized independent risk factors for coronary artery disease. Our results demonstrated the reduction of TG, TC and LDL-C to the patient receiving *S. platensis*. The study that the presence of *S. platensis* reduces the risk of cardiovascular diseases in HIV patients regarding dyslipidemia.

### Limitations of the study

Comprehensive cardiovascular risk factors were assessed in this study. However, the decreased risk of cardiovascular diseases associated with *S. platensis* intake is well known [15], and long term use of first-line HAART may have an impact on cardiovascular systems, which were not assessed in this study. Others factors not controlled in the analysis were the mechanisms of *S. platensis* on lipoprotein lipase which may have influenced the decreased of triglycerides.

## Conclusion

The consumption of balance diet combined with *S. platensis* powder has beneficial effects of lipids profile in the intervention group at the end of the trial. From this, we can assume that a regular consumption of a balanced diet could slow down metabolic disorders of lipids and reinforce the immune defense system in HIV infected antiretroviral naïve patients.
